# Supplementary material for: Investigating the Effectiveness of a Carb-Free Oloproteic Diet in Fibromyalgia Treatment
Source: Nutrients. 2024 May 25;16(11):1620. doi: 10.3390/nu16111620 (PMC11175073; doi:10.3390/nu16111620)
Supplement: Supplementary file 1 [file nutrients-16-01620-s001.zip › nutrients-3004698-supplementary.pdf]

Investigating the Effectiveness of Carb-Free Oloproteic Diet in Fibromyalgia Treatment

Giuseppe Castaldo[1ǂ], *Carmen Marino*[2,3ǂ], *Mariangela Atteno*[1], *Maria D'Elia*[4,5], *Imma Pagano*[1], *Manuela Grimaldi*[3], *Aurelio Conte*[1], *Paola Molettieri*[1], *Angelo Santoro*[3,5], *Enza Napolitano*[2,3], *Ilaria Puca*[1], *Mariangela Raimondo*[1],*Chiara Parisella*[1], *Anna Maria D'Ursi*[1,3,4*], *Luca Rastrelli*[1,3,4*].

AUTHOR ADDRESS

1 NutriKeto_LAB Unisa - "San Giuseppe Moscati" National Hospital (AORN), Contrada Amoretta, 83100 Avellino (AV), Italy. dursi@unisa.it; rastrelli@unisa.it, giuseppecastaldo@yahoo.it.

2 PhD Program in Drug Discovery and Development, Department of Pharmacy, University of Salerno, Via Giovanni Paolo II, 132, Fisciano, 84084 Salerno, Italy cmarino@unisa.it; enapolitano@unisa.it.

3 Department of Pharmacy, University of Salerno, Via Giovanni Paolo II 132, 84084 Fisciano (SA), Italy. cmarino@unisa.it; magrimaldi@unisa.it; asantoro@unisa.it; enapolitano@unisa.it; dursi@unisa.it; rastrelli@unisa.it.

4 NBFC, National Biodiversity Future Center, Palermo 90133, Italy deliameri1989@gmail.com;dursi@unisa.it; rastrelli@unisa.it.

5 Department of Pharmacy, Scuola di Specializzazione in Farmacia Ospedaliera, University of Salerno, Via Giovanni Paolo II, 132, 84084 Fisciano, Italy. asantoro@unisa.it

ǂThese authors contribute equally to the study

* Correspondence: Prof. Anna Maria D'Ursi, Ph.D. Associate Professor, DIFARMA, University of Salerno via Giovanni Paolo II, 132, 84084, Fisciano (SA) Tel +39089969748, e-mail: dursi@unisa.it. Luca Rastrelli, Department of Pharmacy, University of Salerno, Via Giovanni Paolo II 132, 84084 Fisciano (SA), Italy, Tel.:+39 089969766, e-mail address: rastrelli@unisa.it.

Table of content

Table S1. Inclusion and exclusion study criteria

Table S2.Patients clinical history to detect possible disorders before and during dietary treatment. The frequency of the clinical disorders investigated is expressed as a percentage

Table S3. Calculation of the scale of the average symptom calculated as 0: absence, 1:mild symptom, 2:medium symptom; 3:severe symptom

Table S4. Clinical parameters related to serum and urine of fibromyalgia women calculated before (t0) and after 45 (t45) days of diet. The table shows the average value (Mean) and the relative standard deviation (Dev.std),p.value calculated using T-test comparing t0 vs t45.

Table S5 Rhematological scores' Average; standard deviation and p values for each time point performed using T-test

Table S6.. PLS-DA classification of the five different components (comps) based on accuracy, R2, Q2 related to fibromyalgia patients' serum, urine and salivary extract before and after nutritional reagim, carried out by NMR.

Table S7. Specific tissue-organelle dysmetabolism was predicted using the matrix of serum urinary and salivary metabolites through the *Enrichment tool*. The table shows the metabolites responsible for tissue-organelle-specific dysmetabolism (Hits). the p-value (Raw p). The p-value adjusted by Bonferroni correction and the False Discovery Rate (FDR)

Table S8. Predicted enzymes related to fibromyalgia patients serum, urinary and salivary polar extract after 45 days of nutritional treatment. Enzyme are classified according to p-Values (Raw p); adjusted value Bonferroni correction (Holm p) and FDR values.

Figure S1. General workflow

Figure S2. PLS-DA score scatter plot for the 1H NMR data collected in 1D-1H-CPMG spectra for serum and urine and 1D-NOESY for saliva acquired at 600 MHz. Data represent the serum, urine, and saliva profiles from FM1 and FM2 at baseline (t0) and after 45 days of the diet. Cross-validation was performed to validate the separation and reported:0.78%, 0.79%, and 0.62% values, and 0.76, 0.77,0.35 Q2 values of serum, urine and saliva accuracy,

respectively.

Table S1. Inclusion and exclusion study criteria

Inclusion criteria
• Adult women, older than 18 years until pre-menopause;
• Diagnosis of FM performed by the rheumatologist, according to the Rome III criteria of the American College of Rheumatology, revised in 2010;
• Ability to read and sign informed consent;
• Normal kidney function with serum creatinine 1.2 mg/dl and glomerular filtrate 80 ml/min
• Normal liver function with ALT-AST-GGT
**Exclusion criteria**
• Patients with pathologies that prevent following the ketogenetic dietary intervention;
• Patients currently breastfeeding or pregnant;
• Previous or current clinical history of drug or other substance abuse;
• Presence of other inflammatory diseases;
• Renal failure (creatinine;1,4 mg/dl) or filtered glomerular & lt; 80 ml/min
• Severe or medium-severe liver failure > Child B
• Insulin-dependent diabetes (Type 1)
• Ventricular atrium block with QT 0.44 ms
• Cardiac arrhythmias
• Severe or medium-severe heart failure
• Uncontrolled hypokalemia
• Persistent diarrhea or vomiting
• Heart attack or stroke in the last 12 months
• Ongoing neoplasms
• Serious psychiatric disorders

Table S2.Patients clinical history to detect possible disorders before and during dietary treatment. The frequency of the clinical disorders investigated is expressed as a percentage

	FM1				FM2			
	T0		T45		T0		T45	
	Yes	No	Yes	No	Yes	No	Yes	No
Reflux Oesophagitis	68,18%	31,82%	18.18%	81,82%	69,23%	30,77%	15,38%	84,62%
Skin Candidiasis	30,77%	69,23%	0%	100%	22,00%	78%	0%	100%
Hirsutism	40,90%	59,10%	40.90%	59,10%	30,77%	69,23%	46,15%	54%
Acne	13,63%	86,37%	9.09%	90,91%	23,08%	76,92%	92,31%	7,69%
Alopecia	0%	100%	13.63%	86,37%	69,23%	31%	38,46%	61,54%
White Leaks	63,64%	36,36%	18.18%	81,81%	69,23%	30,77%	38,46%	61,54%

Dysentery	49,91%	50,09%	18.18%	81,82%	7,69%	92,31%	38,46%	61,54%
Constipation	45,45%	54,55%	27.27%	72,73%	69,23%	30,77%	23,08%	76,92%

Frequent Cystitis	63,64%	36,36%	9.09%	90,91%	7,69%	92,31%	23,08%	76,92%
Nail Changes	40,91%	59,09%	18.18%	81,82%	46,15%	53,85%	46,15%	53,85%
Insomnia	100%	0%	50%	50%	76,92%	23%	61,54%	38%
Awakening With Refreshment	100%	0%	68.18%	31,82%	100,00%	0%	69,23%	30,77%
Headache	81,82%	18,18%	54.54%	45,46%	100,00%	0%	53,85%	46,15%
Tiredness	100%	0%	72.72%	27,28%	100,00%	0%	53,85%	46,15%
Difficulty Contrating	100%	0%	100%	0%	100,00%	0%	46,15%	54%
Memory Difficulties	100%	0%	100%	0%	100,00%	0%	53,85%	46%
Painful Diuresis	31,81%	68,19%	18.18%	81,82%	100,00%	0,00%	30,77%	69,23%
Frequent Vomiting	13,64%	86,36%	9.09%	90,91%	7,69%	92,31%	7,69%	92,31%
Meteorism	100%	0%	31.81%	68,19%	100,00%	0%	69,23%	30,77%

Table S3. Calculation of the scale of the average symptom calculated as 0: absence, 1:mild symptom, 2:medium symptom; 3:severe symptom

	FM1		FM2	
**Symptoms**	**T0**	**T45**	**T0**	**T45**
Reflux esophagitis	1.46	0.38	1.53	0.53
White losses	0.83	0.21	1.26	0.53
Frequent diarrhea	0.75	0.25	0.95	0.37
Constipation	1.38	0.42	1.53	0.95
Alvo alterno	0.50	0.21	0.63	0.53
Frequent cystitis	1.17	0.25	1.26	0.47
Snoring	0.67	0.25	0.89	0.58
Insomnia	2.46	0.92	2.68	1.68
Awakening not rested	2.96	1.04	2.84	1.74
Migraine/ headache	2.17	0.79	2.05	1.26
Fatigue/fatigue	2,79	1.54	2.95	2.11
Concentration difficulty	2.46	1.33	2.58	1.63
Memory difficulty	2.42	1.42	2.53	1.79
Muscle cramps	2.13	0.75	2.11	1.00
Muscle-tendon pain	3.00	1.38	2.89	1.89

Painful intercourse	1.17	0.75	1.42	0.95
Painful diuresis	0.96	0.29	0.68	0.16
Meteorism	2.54	0.58	2.63	1.32

Table S4. Clinical parameters related to serum and urine of fibromyalgia women calculated before (t0) and after 45 (t45) of diet. The table shows the averagevalue (Mean) and the relative standard deviation (Dev.std),p.value calculated using T-test comparing t0 vs t45.

		FM1			FM2			
**Parameters**		**Mean**	**Dev.std**	**p-value**	**Mean**	**Dev.std**	**p-value**	**Reference value**
Glucose	t0	82,90	4,67		86,46	4,25		mg/dL 70-105
	t45	81,90	9,38	NS	88,23	7,54	NS	
Creatinine	t0	0,70	0,09		0,70	0,09		mg/dL0,57-1,11
	t45	0,72	0,08	0,0005	0,72	0,12	NS	
AST	t0	18,90	6,92		19,00	7,84		IU/L 5-34
	t45	19,81	5,22	0,4568	19,08	5,78	NS	
ALT	t0	19,95	14,23		23,92	15,79		IU/L 0-55
	t45	22,10	10,07	0,0114	22,08	12,40	NS	
GGT	t0	25,00	40,98		20,85	11,99		IU/L 9-36
	t45	15,43	16,13	NS	17,08	9,69	NS	
Col tot	t0	206,60	32,76		208,85	32,73		mg/dL Optimal≤ 200
	t45	191,50	44,97	0,0046	174,46	28,04	NS	
HDL	t0	66,62	15,98		55,85	11,43		mg/dL Optimal: superior 65<br>Moderate risk:45-65<br>Hight risk: ≤ a 45
	t45	51,33	13,36	6,69E-07	46,85	8,46	0,03	
LDL	t0	131,50	32,22		139,23	23,67		mg/dL Optimal: 130<br>Moderate Risk :130-159<br>Hight Risk: superior a 160
	t45	123,90	44,05	0,0026	112,54	26,57	0,01	
Tryglicerid	t0	109,80	73,33		92,00	32,30		mg/dL Optimal≤ 200
	t45	80,43	31,40	0,00004	65,62	18,72	0,02	
Transferrine	t0	329,00	58,94		291,15	27,52		mg/dL Optimal 180-382
	t45	295,50	70,19	8,44E-06	264,62	32,20	0,03	
Folic Acid	t0	6,17	3,20		7,05	4,15		ng/mL<br>2,80-12,40
	t45	7,70	2,66	7,08E-05	9,40	4,49	NS	
Vitamin B12	t0	401,90	132,91		552,58	274,19		pg/mL<br>187-883
	t45	461,00	197,04	3,30E-05	656,46	425,68	NS	
Insulinemia	t0	7,34	3,36		8,38	7,67		mcUI/mL<br>0,0-25,0
	t45	5,10	2,65	0,0186	5,96	3,51	NS	
Cortisol	t0	8,64	3,39		5,81	1,54		µg/dL

	t45	8,69	3,41	0,0411	6,92	2,68	NS	3,7-19,4
Fibrinogen	t0	327,30	76,38		355,00	81,29		mg 180-250
	t45	365,00	61,46	0,0084	379,08	80,77	NS	
HB	t0	12,60	0,78		13,57	1,44		g/dL<br>13,0-17,0
	t45	12,89	0,88	3,31E-06	13,46	1,24	NS	
HCT	t0	36,83	2,20		40,29	4,21		%<br>38,0-49,0
	t45	37,52	2,60	0,00013	38,99	2,69	NS	
Urine pH	t0	5,76	0,51		5,62	0,30		pH<br>5,0-6,5
	t45	3,53	0,79	NS	5,88	0,74	NS	
HOMA	t0	1,51	0,61		1,37	0,31		mg/dL 0,23 – 2,5
	t45	1,06	0,64	0,032	1,07	0,66	0,03	
Uric acid	t0	4,30	1,59		4,44	0,70		mg/dL 2,6-6,0
	t45	4,58	1,50	9,29E-05	4,72	1,08	NS	
HTSH	t0	1,30	0,70		1,064	0,69		µIU/mL <6 months :0,3700-5,5400<br>6mm-14aa:0,6100-4,4300<br>15-19anni:0,2500-3,4500<br>Adults :0,3500-4,9400
	t45	1,23	0,76	0,017	0,966	0,62	NS	
FT3	t0	2,81	0,44		2,838	0,87		pg/mL<br><1anno:2,23-5,19<br>1-12anni:2,74-4,49<br>13-15anni:2,43-4,02<br>16-19anni:2,01-3,59<br>Adults1,71-3,71
	t45	2,47	0,31	NS	2,687	0,32	NS	
FT4	t0	0,95	0,10		0,995	1,71		ng/dL<br>5-14giorni:0,87-3,35<br>15-29giorni:0,55-2,65<br>30gg-1anno:0,83-1,84<br>2-19anni:0,86-1,39<br>Adults:0,70-1,48
	t45	1,01	0,14	0,0069	1,057	0,27	NS	
C protein	t0	0,61	0,61		0,36	1,49		mg/dL ≤0,50
	t45	0,40	1,06		0,25	0,45	NS	
25 OHD	t0	28,90	1,26		35,77	1,27		ng/mL<br>DEFICIENCIES:fino a 10<br>INSUFFICIENCY:11-30<br>SUFFICIENCY:31-100<br>TOXICITY’:<100
	t45	42,57	1,27	4,12E-02	34,46	0,52	NS	
testosterone	t0	30,81	9,02		26,92	11,59		ng/dL<br><15(7-9anni)<br>2-42(10-11anni)
	t45	29,67	11,70	1,18E-05	31,31	138,41	NS	

								6-64(12-13anni)<br>9-49(14-15anni)<br>8-63(16-17anni)<br>10-59(18-50anni)<br>6-25(<51anni)
estradiol	t0	92,95	94,34		112,83	149,58		pg/mL<br>21-251 follicolar<br>38-649 pick<br>21-312 luteinic<br>10-28 postmaenopause
	t45	116,00	130,79	NS	107,62	63,27	NS	
SHBG	t0	146,50	160,74		81,31	4,15		nmol/L
	t45	175,80	172,68	6,44E-12	93,85	4,49	NS	18-114

ALT: Alanine aminotransferase; AST: Aspartate amminotransferase; GGT: Gamma glutamil transferase ;HDL: High density lipoprotein ;LDL: Low density lipoprotein; HTSH: Thyroid-Stimulating Hormone; FT3: tri-iodothyronine; FT4: thyroxine;25 OHD: calcidiol; SHGB: Sex hormone binding globulin; HB: hemoglobin; HCT: Hematocrit; HOMA: Homeostasis Model Assessment

Table S5 Rhematological scores' Average; standard deviation and p values for each time point performed using T-test

		FM1				FM2				FM1 vs FM2	
		Average	dev std	% change respect to t0	p-value FM1 t0 vs t45	Average	dev std	% change respect to t0	p-value FM2 t0 vs t45	p-value	
WPI	t0	15,46	3,97		1.83E-11	14,44	3,22		0,02	FM1 t0 vs FM2 t0<br>WPI:NS<br>SSS:NS<br>HAM-A:NS<br>HAM-D:NS	FM1 t45 vs FM2 t45:<br>WPI:0.03<br>SSS:0.01<br>HAM-A:0.002<br>HAM-D:0.004
	t45	5,70	3,59	-63,15		10,74	5,39	-25,67			
SSS	t0	9,33	1,71		7.28E-09	9,44	1,82		0,07		
	t45	5,35	2,21	-42,7		7,05	2,52	-25,33			
HAM-A	t0	28,50	8,44		4.13E-11	28,58	8,70		0,04		
	t45	11,18	5,13	-60,76		20,58	10,12	-28			
HAM-D	t0	19,21	5,27		1.73E-06	17,89	4,04		0,07		
	t45	12,27	6,16	-36,1		14,89	4,02	-16.8			

N.S no significant

Table S6. PLS-DA classification of the five different components (comps) based on accuracy, R2, Q2 related to fibromyalgia patients' serum, urine and salivary extract before and after nutritional reagim, carried out by NMR.

MEASURE SERUM	1 COMP	2 COMP	3COMP	4COMP	5COMP
**Accurancy**	0.77	1.00	1.00	1.00	1.00
**Q2**	0.92	0.97	0.99	0.99	0.99
**R2**	0.96	0.97	0.96	0.96	0.96
**MEASURE URINE**	**1 COMP**	**2 COMP**	**3COMP**	**4COMP**	**5COMP**
**Accurancy**	0.65	0.98	0.98	0.96	0.95

**Q2**	0.86	0.97	0.98	0.98	0.98
**R2**	0.84	0.94	0.94	0.92	0.91
MEASURE SALIVA	**1 COMP**	**2 COMP**	**3COMP**	**4COMP**	**5COMP**
**Accurancy**	0.64	0.77	0.72	0.68	0.67
**Q2**	0.87	0.80	0.84	0.88	0.89
**R2**	0.60	0.63	0.71	0.54	0.52

Table S7. Specific tissue-organelle dysmetabolism was predicted using the matrix of serum urinary and salivary metabolites through the *Enrichment tool*. The table shows the metabolites responsible for tissue-organelle-specific dysmetabolism (Hits). the p-value (Raw p). The p-value adjusted by Bonferroni correction and the False Discovery Rate (FDR)

	FM1				*FM2*			
	t0vst45				*t0vst45*			
**Serum**	Hits	Raw p	Holm p	FDR	Hits	Raw p	Holm p	FDR
Skeletal Muscle	18	1,60E-25	5,29E-24	1,36E-24	12	1,06E-05	1,80E-04	2,53E-05
Muscle	22	1,89E-25	6,04E-24	1,36E-24	16	5,91E-06	1,06E-04	1,60E-05
Spleen	15	3,39E-25	1,05E-23	2,04E-24				
Pancreas	16	1,20E-23	3,59E-22	6,15E-23	13	1,13E-05	1,80E-04	2,53E-05
Brain	16	1,13E-17	3,15E-16	4,50E-18				
Mitochondria	12	4,90E-16	1,28E-14	1,61E-15				
Intestine	17	1,23E-13	3,07E-12	3,68E-13	13	3,51E-07	7,37E-06	1,35E-06
Liver					19	2,46E-07	5,66E-06	1,15E-06
Urine	Hits	Raw p	Holm p	FDR	Hits	Raw p	Holm p	FDR
Muscle	15	1,85E-16	5,18E-15	5,18E-15	15	6,74E-06	1,62E-04	3,77E-05
Bladder	17	0.00078261	0.0086087	0.0012174	17	1,51E-05	3,32E-04	6,04E-05
Spleen	12	0.00088022	0.0088022	0.0012972	12	2,77E-05	5,83E-04	9,71E-06
Intestine	12	0.0026004	0.023404	0.0036406				
Pancreas	13	0.0031641	0.025313	0.0042188	13	3,46E-05	6,91E-04	1,08E-04
Saliva	Hits	Raw p	Holm p	FDR				
Skeletal Muscle	12	1,47E-13	3,39E-12	6,87E-13				
Muscle	18	5,13E-07	1,44E-05	3,87E-06				
Liver	17	6,25E-07	1,69E-05	3,87E-06				
Intestine	16	3,61E-05	9,03E-04	1,60E-04				

Table S8 Predicted enzymes related to fibromyalgia patients serum, urinary and salivary polar extract after 45 days of nutritional treatment. Enzyme are classified according to p-Values (Raw p); adjusted value Bonferroni correction (Holm p) and FDR values.

**FM1**			
**Serum**	**Raw p**	**Holm p**	**FDR**
Trehalose exchange	2.67E-17	5.07E-15	5.07E-15
N-acetyl-glucosamine lysosomal efflux	2.37E-15	4.45E-13	1.12E-13
N-acetylglucosamine kinase	2.37E-15	4.45E-13	1.12E-13

glutamine synthetase	4.08E-15	7.59E-13	1.55E-14
ATP synthase (four protons for one ATP)	4.92E-15	9.11E-13	1.56E-13
glyceraldehyde-3-phosphate dehydrogenase	1.49E-13	2.75E-11	4.05E-12
Glutamate transport via Na H symport and K antiport	2.81E-13	5.14E-11	6.67E-12
pyruvate carboxylase	1.29E-10	2.25E-08	1.4E-09
**Urine**	**Raw p**	**Holm p**	**FDR**
pyruvate carboxylase	8.2E-19	1.31E-16	1.01E-16
Trehalose exchange	1.27E-18	2.02E-16	1.01E-16
glutamine synthetase	6.4E-18	1.01E-15	3.41E-16
glyceraldehyde-3-phosphate dehydrogenase	1.73E-16	2.72E-14	6.92E-15
Glutamate transport via Na H symport and K antiport	1.66E-15	2.6E-13	5.32E-14
L-Phenylalanine exchange	3.26E-14	5.02E-13	7.46E-13
ATP synthase (four protons for one ATP)	1.31E-13	1.99E-11	2.32E-12
**Saliva**	**Raw p**	**Holm p**	**FDR**
phosphoglycerate dehydrogenase	4.16E-02	0.0075333	0.00025329
phosphoserine phosphatase (L-serine)	4.16E-02	0.0075333	0.00025329
phosphoserine transaminase	4.16E-02	0.0075333	0.00025329
Trehalose exchange	7.36E-01	0.013093	0.00043522
glutamine synthetase	0.00015047	0.026633	0.00086622
glyceraldehyde-3-phosphate dehydrogenase	0.00024613	0.042827	0.0013107
**FM2**			

**Serum**	Raw p	Holm p	FDR
citrate synthase	1,14E-07	1,21E-05	3,14E-06
pyruvate carboxylase	1,15E-07	1,22E-05	3,14E-06
glutamine synthetase	3,63E-06	3,81E-04	7,92E-05
N-acetyl-glucosamine lysosomal efflux	6,78E-06	7,05E-04	1,06E-04
N-acetylglucosamine kinase	6,78E-06	7,05E-04	1,06E-04
glucosamine-6-phosphate deaminase	1,28E-05	1,30E-03	1,74E-04

Figure S1. General workflow

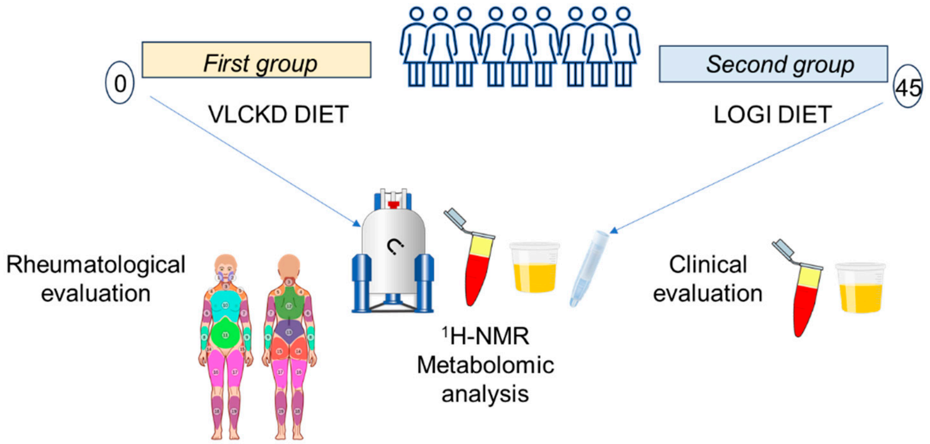

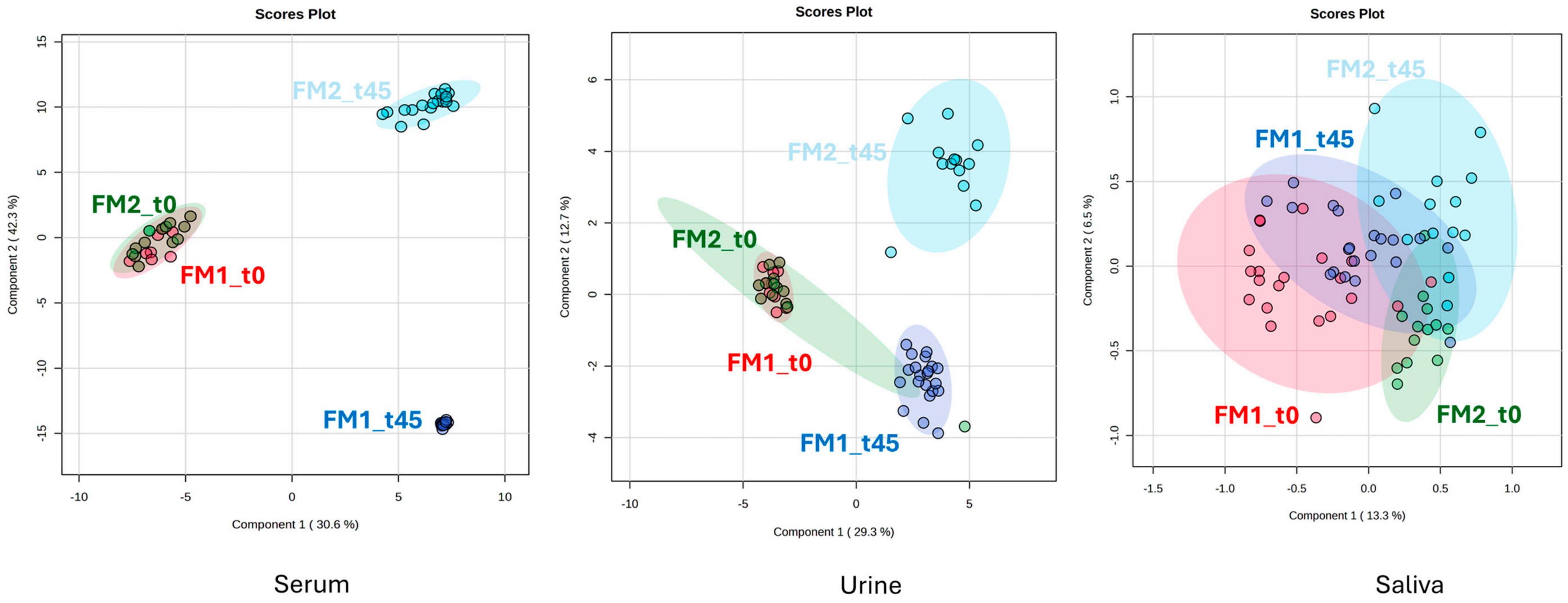

Figure S2. PLS-DA score scatter plot for the ^{1}H NMR data collected in 1D-^{1}H-CPMG spectra for serum and urine and 1D-NOESY for saliva acquired at 600 MHz. Data represent the serum, urine, and saliva profiles from FM1 and FM2 at baseline (t0) and after 45 days of the diet.
Cross-validation was performed to validate the separation and reported:0.78%, 0.79%, and 0.62% values, and 0.76, 0.77,0.35 Q2 values of serum, urine and saliva accuracy, respectively.
